# Infection with *Toxoplasma gondii* triggers coagulation at the blood-brain barrier and a reduction in cerebral blood flow

**DOI:** 10.1186/s12974-024-03330-1

**Published:** 2025-01-08

**Authors:** Evelyn M. Hoover, Christine A. Schneider, Christian Crouzet, Tatiane S. Lima, Dario X. Figueroa Velez, Cuong J. Tran, Dritan Agalliu, Sunil P. Gandhi, Bernard Choi, Melissa B. Lodoen

**Affiliations:** 1 Department of Molecular Biology and Biochemistry, University of California Irvine, Irvine, 92697 USA; 2 Institute for Immunology, University of California Irvine, Irvine, 92697 USA; 3 Department of Biomedical Engineering, University of California Irvine, Irvine, 92697 USA; 4 Beckman Laser Institute and Medical Clinic, University of California Irvine, Irvine, 92697 USA; 5 Department of Biological Sciences, California State Polytechnic University, Pomona, CA 91768 USA; 6 Department of Neurobiology and Behavior, University of California Irvine, Irvine, 92697 USA; 7 Center for the Neurobiology of Learning and Memory, University of California Irvine, Irvine, 92697 USA; 8 Department of Surgery, University of California Irvine, Irvine, 92697 USA; 9 Edwards Lifesciences Foundation Cardiovascular Innovation Research Center, University of California Irvine, Irvine, 92697 USA; 10 Department of Pathology and Cell Biology, Columbia University Irving Medical Center, New York, NY 10032 USA; 11 Department of Neurology, Columbia University Irving Medical Center, New York, NY 10032 USA

**Keywords:** *Toxoplasma gondii*, CNS infection, Thrombosis, Cerebral blood flow, Blood-brain barrier

## Abstract

**Background:**

Immunothrombosis is the process by which the coagulation cascade interacts with the innate immune system to control infection. However, the formation of clots within the brain vasculature can be detrimental to the host. Recent work has demonstrated that *Toxoplasma gondii* infects and lyses central nervous system (CNS) endothelial cells that form the blood-brain barrier (BBB). However, little is known about the effect of *T. gondii* infection on the BBB and the functional consequences of infection on cerebral blood flow (CBF) during the different stages of infection.

**Main body:**

We demonstrate that brain endothelial cells upregulate the adhesion molecules ICAM-1 and VCAM-1 and become morphologically more tortuous during acute *T. gondii* infection of mice. Longitudinal two-photon imaging of cerebral blood vessels during infection in mice revealed vascular occlusion in the brain, prompting an analysis of the coagulation cascade. We detected platelet-fibrin clots within the cerebral vasculature during acute infection. Analysis of CBF using longitudinal laser-speckle imaging during *T. gondii* infection demonstrated that CBF decreased during acute infection, recovered during stable chronic infection, and decreased again during reactivation of the infection induced by IFN-γ depletion. Finally, we demonstrate that treatment of mice with a low-molecular-weight heparin, an anticoagulant, during infection partially rescued CBF in *T. gondii*-infected mice without affecting parasite burden.

**Conclusions:**

Our data provide insight into the host-pathogen interactions of a CNS parasite within the brain vasculature and suggest that thrombosis and changes in cerebral hemodynamics may be an unappreciated aspect of infection with *T. gondii*.

**Supplementary Information:**

The online version contains supplementary material available at 10.1186/s12974-024-03330-1.

## Background

Hemostasis is the physiological response to vessel injury, in which the coagulation cascade is activated to form a thrombus or blood clot, consisting of aggregated platelets and cross-linked fibrin [1]. During blood vessel injury, clotting can be initiated via the extrinsic pathway when subendothelial tissue factor is exposed to coagulation factors in the blood, resulting in activation of factor Xa, which cleaves prothrombin to thrombin [1]. Thrombin then cleaves fibrinogen to fibrin. At sites of vessel damage, the formation of a fibrin-platelet clot is critical to prevent hemorrhage. Although tissue factor is typically found on subendothelial cells (i.e., astrocytes in the brain) [2], it can also be expressed on myeloid cells during inflammation, and potentiate immunothrombosis [3–6]. Clotting can also be initiated in response to negatively charged molecules and surfaces (i.e., DNA, RNA, plasma membrane of activated platelets), which activate factor XII in the blood via the intrinsic pathway [7].

Thrombosis is considered to be a pathological manifestation of hemostasis and occurs when thrombi form within blood vessels, causing vessel occlusion [8]. The infection of individuals with diverse pathogens has been linked to increased risk of blood clots during infection, including in those infected with viruses (e.g., HIV, influenza, SARS-CoV2), blood-borne bacteria (e.g. *E. coli*, *Yersinia enterocolitica*), and eukaryotic parasites (*Plasmodium falciparum*) [4, 9–11]. In some cases, clotting can be beneficial to the host, helping to sequester the pathogen and limit its dissemination [12]. However, when thrombosis occurs within the brain vasculature during infection, it can be associated with negative outcomes, such as in pediatric patients with cerebral malaria [13–16], or in COVID-19 adult patients [17]. Additionally, despite the highly vascularized nature of the brain, thrombi in the brain can negatively affect cerebral blood flow (CBF) and brain function, leading to irreversible neuronal loss and neurological sequelae, as seen in stroke patients [18]. Indeed, patients with sepsis and COVID-19 have been found to have reduced CBF [11, 17, 19].

*Toxoplasma gondii* is a eukaryotic protozoan parasite that is found worldwide [20] and that infects and lyses brain microvascular endothelial cells [21] prior to parasite entry to the brain. There is also evidence that the cerebral microvasculature is perturbed during *T. gondii* infection [22–25]. However, less is known about the effect of *T. gondii* infection on the blood-brain barrier and the functional consequences of infection on CBF during the different stages of infection: (1) the acute stage in which *T. gondii* replicates in the peripheral organs and enters the brain from the periphery; (2) the stable chronic stage, in which the parasites are located within cysts in the brain and muscle tissue; and finally, (3) the reactivation stage, which occurs in immunocompromised patients when the latent infection is no longer controlled [26, 27].

Here we used intravital imaging approaches coupled with fixed tissue analysis in mice to demonstrate that brain endothelial cells are activated during acute infection with *T. gondii*, upregulated expression of adhesion molecules ICAM-1 and VCAM-1, and became morphologically more tortuous during infection. We also detected vascular occlusion in the brain and the formation of platelet-fibrin clots within the cerebral vasculature during acute *T. gondii* infection. Longitudinal laser speckle imaging revealed that CBF decreased during acute infection, recovered during stable chronic infection, and decreased again during reactivation of the infection. Finally, we demonstrate that treatment with a low-molecular-weight heparin, nadroparin, partially rescued CBF in *T. gondii*-infected mice. These data suggest that changes in cerebral hemodynamics may be an underappreciated feature of systemic inflammation and pathogen infection.

## Materials and methods

### Animals

All procedures and protocols were approved by the Institutional Animal Care and Use Committee (IACUC) at the University of California, Irvine. Procedures were performed on 2- to 6-month-old male and female mice. Wild-type (WT) C57BL/6J mice were purchased from Jackson Laboratories. Heterozygous Tie-2::eGFP-Claudin-5 transgenic mice were bred in-house with WT C57BL/6J mice and screened by PCR as previously published [28].

### Acute and chronic infections and reactivation

Mice were intraperitoneally (i.p.) injected with 1-5 × 10^4^*T. gondii* tachyzoites for acute infection experiments and 200 tachyzoites for chronic infection experiments in 200 µL of PBS. GFP- or tdTomato-expressing type II *Prugniaud* parasites were used. Tachyzoites were serially passaged and maintained in human foreskin fibroblasts as described previously [29]. Mock-infected control mice were injected i.p. with 200 µL of sterile PBS (Corning). Reactivation was induced by i.p. injection of 2 mg of anti-IFN-γ (XMG1.2, Bio X cell) or rat IgG1 isotype control (Bio X cell) per mouse at 28 and 32 dpi. At experimental endpoints, mice were sedated with 2.5% Tribromoethanol (Avertin; Sigma-Aldrich) and perfused transcardially with 30 mL 1× PBS (Corning) to remove non-adherent blood cells, as previously reported [30].

### Parasite quantification

For B1 quantification, the Blood and Tissue kit (Qiagen) was used to extract genomic DNA from 10 mg of homogenized brain tissue following the manufacturer’s guidelines. For SAG1 quantification, total RNA was harvested from 10 mg of homogenized brain tissue using the RNeasy Kit (Qiagen) and treated with DNase I (Life Technologies) to remove contaminating genomic DNA. cDNA was synthesized using the Superscript III First-Strand Synthesis kit (Life Technologies), according to the manufacturer’s instructions, and subsequently used as a template in quantitative real-time PCR (qPCR). qPCR was performed in triplicate using a Bio-Rad iCycler PCR system and iTaq Universal SYBR Green Supermix (Bio-Rad). All primers were synthesized by Integrated DNA Technologies. Primer pairs for B1: CAGATGTGCTAAAGGCGTCA (sense), GCCCTAGACAGACAGCGAAC (anti-sense). B1 qPCR data from genomic DNA were used to determine parasite concentration (parasites/mg) by referencing a standard curve of parasites as previously published [31]. GAPDH primers were previously published [32]. Primers for SAG1: CAGCACTCTTGGTCCTGTCA (sense), TGGCACCATTATCACTCGAA (anti-sense). qPCR data were analyzed using the threshold cycle method [33], and gene expression data are shown normalized to that of the housekeeping gene GAPDH. In all RT-qPCR assays, cDNA generated in the absence of reverse transcriptase, as well as water in place of the DNA template, were used as negative controls, and these samples were confirmed to have no amplification.

### Immunofluorescence staining, imaging, and analysis

Brains were collected and incubated in 4% PFA for 6–16 h, cryopreserved with 30% sucrose in 1x PBS until the tissues sunk, and embedded in OCT freezing medium (Thermo Fisher Scientific). After sectioning, tissue was stored in cryoprotection media (30% sucrose, 30% ethylene glycol, 0.1 M Phosphate buffer, 1% PVP-40) at -20 °C. Nuclei were stained using Hoechst dye (1:20,000; Sigma) just prior to the final wash. For detection of ICAM-1 and VCAM-1, 16-µm-thick sections were blocked and permeabilized in IFA buffer [1X PBS (Corning), 5% normal donkey serum (Southern Biotech, Birmingham, AL), 0.3% Triton-x 100 (Thermo Fisher Scientific)] for 2–4 h. Antibodies against GLUT1 (1:400; Millipore), ICAM-1 (1:100; Biolegend), or VCAM-1 (1:100; Biolegend) in IFA buffer were incubated with sections for 48 h. Secondary antibodies (1:500) were incubated with sections for 24 h. Z-stacks were taken with a 63X objective on a confocal Leica SP8 microscope (Leica Microsystems). Ten random non-adjacent fields of view (FOVs) in the cortex were imaged. For detection of CD41 and fibrin, 100-µm-thick sagittal sections were blocked and permeabilized in IFA buffer for 16 h. Antibodies against GLUT1 (1:400; Millipore), fibrin (1:200; Thermo Fisher Scientific), or CD41 (1:100; Biolegend) in IFA buffer were incubated with sections for 72 h. Secondary antibodies (1:500) were incubated with sections for 48 h. Immediately prior to imaging, brain tissues were cleared using the ultrafast optical clearing method [34]. A widefield (pinhole set to 3 Airy Units) tilescan was imaged with a 20X objective on a Leica SP8 microscope.

The quantification of ICAM-1 and VCAM-1 was done on max z-stack projections. The quantification of CD41 and fibrin was done on ten random 300 × 300 μm FOVs throughout the cortex from widefield (pinhole set to 3 Airy Units) fluorescence tilescans of 100 μm-thick sagittal sections. We used FIJI, a processing package based on ImageJ2 for all of our image processing [35]. Images were first globally smoothed using FIJI’s smooth function, the channels were split, and a global threshold was applied based on maximizing signal to noise to determine areas that were positive for GLUT1 (to denote vessels), ICAM-1, VCAM-1, CD41, or fibrin. Afterward, the percent positive areas of ICAM-1, VCAM-1, CD41, or fibrin within the GLUT1 positive areas were calculated.

### Serum cytokine analysis

Whole blood was collected into coagulation tubes (BD Biosciences) prior to transcardial perfusion [30]. Coagulated blood was centrifuged per the manufacturer’s instructions to isolate sera. Cytokines were measured using the LegendPlex bead-based assay (Biolegend) per kit instructions at a dilution of 1:2. Samples were analyzed on a Novocyte flow cytometer (Agilent). IFN-γ was measured using the ELISA MAX Deluxe Set (BioLegend), according to the manufacturer’s instructions.

### 2-Photon imaging

In Tie-2::eGFP-Claudin-5 heterozygous mice, a cranial window was installed by excising a portion of the skull and attaching a coverslip in its place at 1–2 weeks prior to imaging, as previously described [28, 30, 36]. Imaging was performed using a resonant 2-photon microscope (Neurolabware) equipped with an Olympus 20 × (0.8 NA) water-immersion objective. A Ti: Sapphire laser tuned to 900 nm (Mai-Tai HP, SpectraPhysics) was used to stimulate eGFP fluorescence. Emission was filtered using a 510/84-nm BrightLine bandpass filter (Semrock) and acquired using Scanbox acquisition software. An electrically tunable lens (Optotune) was used for volumetric scanning over 1–2 min in 5–6 fields. To minimize motion artifacts, mice were anesthetized with Isoflurane (Patterson Veterinary) in O_2_ (2% for induction and 1-1.5% for maintenance). Sterile eye ointment (Rugby) was applied to prevent corneal drying, and body temperature was maintained at 37˚C with a custom-made closed loop heating pad. At the end of each imaging session, mice were injected intravenously via the tail vein with 100 µL 0.5% Biocytin-TMR (Invitrogen) in sterile 1x PBS to visualize vascular perfusion. For image processing, focal planes were sum binned and motion corrected using HyperstackReg in FIJI.

### Endothelial cell morphology analysis

Day 0 (baseline, prior to infection) and day 6 imaging was performed by 2-photon microscopy of eGFP-Claudin-5 mice injected with PBS (control) or infected with *T. gondii.* Endothelial cells were compared in each FOV, and cells within the same FOV at both day 0 and day 6 post-infection were analyzed. Endothelial cell borders delineated by eGFP-Claudin-5 were hand-traced in FIJI using a stylus and tablet. Cell area and perimeter were assessed using the measure function, and the shape index was calculated using the standard formula: $\:\text{s}\text{h}\text{a}\text{p}\text{e}\:\text{i}\text{n}\text{d}\text{e}\text{x}=\frac{4{\uppi\:}\text{*}\text{a}\text{r}\text{e}\text{a}}{{\left(\text{p}\text{e}\text{r}\text{i}\text{m}\text{e}\text{t}\text{e}\text{r}\right)}^{2}}$, as has previously been published [37].

Endothelial cell protrusions were counted at day 0 and day 6 along claudin-5 cell borders. Protrusions were defined as sections of cell border with a visible open center less than 8 μm in diameter. The same cells were quantified at each time point. A minimum of 3 vessel sections was quantified for each mouse across at least three fields of view (*n* = 32–52 cells total per mouse).

### Laser speckle imaging

Surgeries and laser speckle imaging (LSI) in mice were performed as previously published [38]. In brief, mice were anesthetized with isoflurane (2% for induction and 1.5% for maintenance, Patterson Veterinary). The scalp was resected, and cyanoacrylate was applied to the skull. Finally dental cement was placed around the cyanoacrylate to provide a well over which a cover slip could be applied. Mice recovered from surgery for at least two weeks prior to infection. LSI to measure cerebral blood flow (CBF) was performed using a long-coherence 785 nm laser. Raw speckle images (10 ms exposure time) were acquired using a 4x Achrovid objective with a 37 mm working distance (Edmund Optics). Cross-polarization optics were used to remove specular reflection.

To measure the changes in CBF during acute infection, we imaged mice at 0, 4, and 7 dpi. For the chronic infection and reactivation experiments, mice were imaged at 0, 7, 14, 28, 34, and 38 dpi. During LSI, mice were anesthetized with O_2_ vaporized isoflurane in the same way as for the 2-photon imaging, and body temperature was maintained at 37˚C with a feedback heating pad (Harvard Apparatus, Holliston, MA). As in Hoover et al., CBF was calculated using a simplified speckle imaging equation $\:\text{C}\text{B}\text{F}=\frac{1}{2T{K}^{2}}$, where T is camera exposure time and K is the measured speckle contrast, and two semi-elliptical regions of interest (ROIs) were used to obtain longitudinal CBF data from each hemisphere [39]. As in our prior study, we selected the ROIs to measure the largest area of each hemisphere, while excluding artifact from the skull sutures (coronal, sagittal, and lambdoid) as well as the dental cement around the cyanoacrylate, which falsely depress the CBF calculation. ROIs were also selected for regions where we could consistently measure the same size FOV for every animal using our surgical technique. The median CBF from each ROI was calculated, and the median CBF values for the right and left hemispheres were averaged. Relative CBF (rCBF) was calculated as a percentage of baseline CBF (set to 100%).

### Low-molecular-weight heparin treatment

For the experiments involving heparin treatment, mice were given subcutaneous injections of 100 units/kg of nadroparin calcium, a low-molecular-weight heparin, in PBS (Sigma-Aldrich) twice daily, roughly twelve hours apart. Control animals received the same volume of PBS only. Treatment was started at 0 dpi after LSI imaging and continued until 7 dpi when mice were euthanized.

### Fibrin extraction and western blotting

Insoluble (fibrin-containing) protein was extracted from liver tissue as described by Cortes-Canteli et al. [40]. Frozen liver tissue was homogenized in 5 volumes (g: mL) of RIPA lysis and extraction buffer (Thermo Fisher Scientific) containing protease inhibitors. The homogenate was centrifuged at 4 °C at 10,000 g for 10 min. The supernatant (soluble fraction) was discarded, and the process was repeated twice more. To extract the insoluble fraction (fibrin-containing fraction), the pellet was homogenized in 3 M urea, mixed for 2 h at 37 °C, and centrifuged at 14,000 g for 15 min. The pellet was resuspended in SDS loading buffer and incubated at 65 °C for 30 min.

After protein quantification, 20 µg of insoluble liver protein was analyzed by Western blotting using the following antibodies: mouse monoclonal anti-fibrin antibody (59D8, Millipore) and mouse monoclonal anti-β-actin antibody (C4, Santa Cruz). Peroxidase-conjugated secondary antibodies were used (Cell Signaling). Membranes were developed using ECL (Thermo Scientific) and detected using a ChemiDoc imaging system (BioRad). Quantitative analysis of blots was performed using ImageJ, and β-actin was used as a loading control. The results were expressed as a percentage of the value for the negative control (Mock-infected, PBS-treated group).

### Flow cytometry

Livers were processed to single-cell suspensions using enzymatic digestion with collagenase diluted in Hank´s balanced salt solution, followed by ACK lysis buffer (Life Technologies). Brains were processed to single-cell suspensions using enzymatic digestion with Dispase II (Roche Applied Science) diluted in Hepes-buffered saline, followed by myelin removal using a Percoll gradient (GE Healthcare). Blood was removed via cardiac puncture into EDTA anticoagulant tubes (Greiner Bio-one). Red blood cells were lysed using ACK lysis buffer (Life Technologies). Liver, brain, or blood cells were resuspended in staining buffer (1× PBS + 3% FBS) with 10% TrueStain FcX (Biolegend) to block nonspecific antibody binding to Fc receptors. Cells were surface-stained with directly conjugated antibodies (anti-CD45-bv785, anti-CD11b-BV605, anti-Ly6G-BV510, anti-Ly6C-PerCP/Cy5.5, anti-CD19-PE/Cy7, and anti-CD3-APC/Cy7). Samples were analyzed on a NovoCyte flow cytometer (Agilent), and the data were analyzed using FlowJo software (Treestar).

### Statistics

GraphPad Prism 8.0.1 software was used for statistical analyses. When only two non-longitudinal groups were compared, an unpaired Student’s *t*-test was used to test for significance. A one-way ANOVA with a post-hoc Tukey test was used to test for significance in the serum cytokine analysis. For the endothelial cell shape analysis, a paired Student’s *t*-test was used to test for significance. For the acute and chronic infection, endothelial cell protrusion analysis, CBF, and weight loss experiments, significance was tested with a repeated measures two-way ANOVA, with a post-hoc Sidak’s multiple comparisons test. For the reactivation and nadroparin-treatment experiments, CBF and weight loss significance was calculated with a mixed-effects analysis with a post-hoc Sidak’s multiple comparisons test.

## Results

### Effect of acute *T. gondii* infection on CNS vascular endothelium

**Fig. 1:**
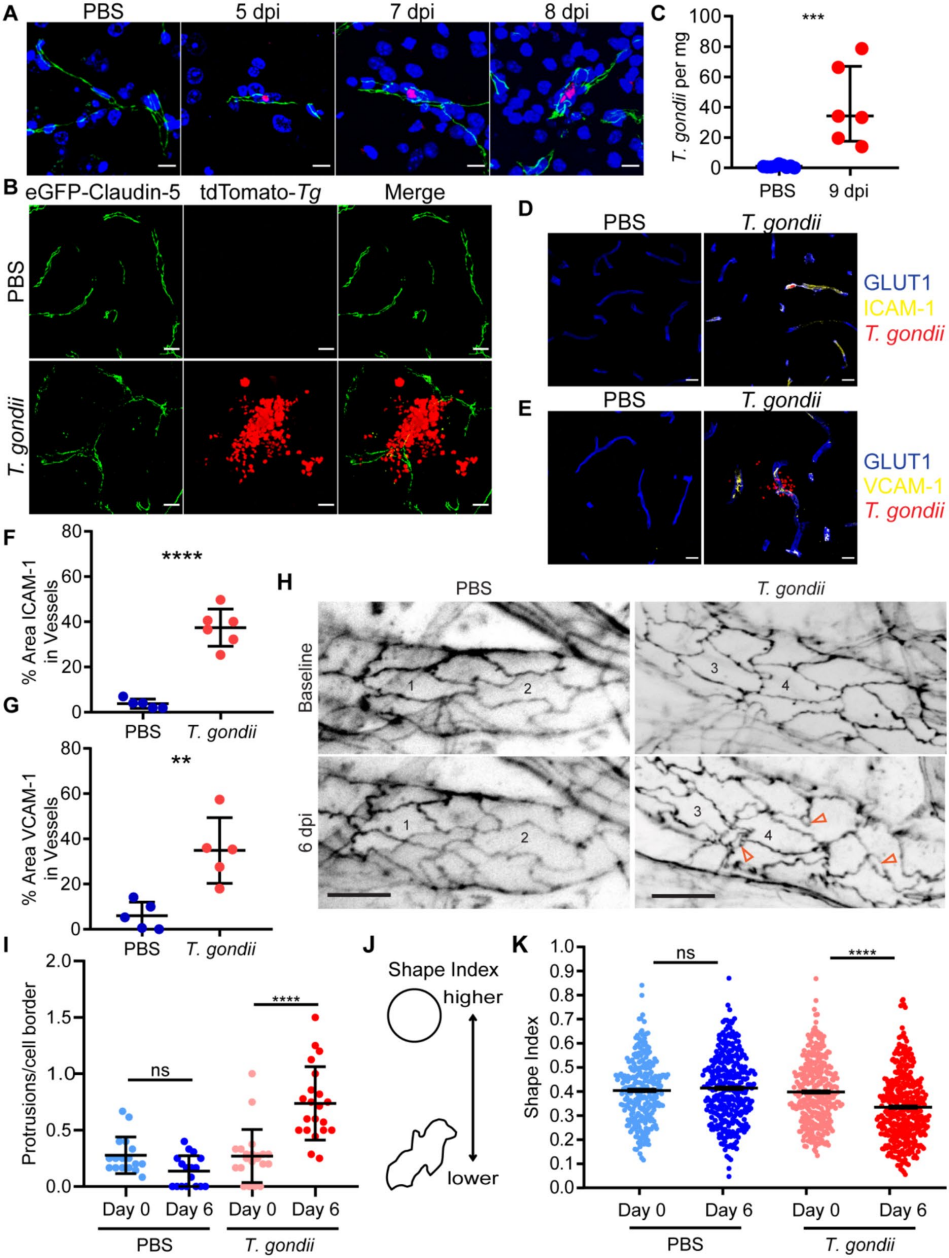
Endothelial cell activation in response to *T. gondii* infection. **A**) Representative confocal microscopy of brain sections from eGFP-Claudin-5 mice injected with PBS or *T. gondii* at 5, 7, and 8 days post-infection (dpi). Sections stained with Hoechst (blue) and imaged for eGFP-Claudin-5 (green), and *T. gondii* (red). Scale bars, 10 μm. **B**) Confocal microscopy of brain sections from PBS-injected or *T. gondii*-infected eGFP-Claudin-5 mice at 9 dpi. Scale bars, 15 μm. **C**) B1 qPCR of DNA extracted from brain homogenates to determine *T. gondii* per mg of brain tissue in mice injected with PBS or *T. gondii* (9 dpi). Each circle represents one mouse. **D**-**E**) Representative confocal images of brain sections stained with antibodies against GLUT1 and (**D**) ICAM-1 or (**E**) VCAM-1 in C57BL/6J mice injected with PBS or *T. gondii* at 7 dpi. Scale bars, 15 μm. **F**-**G**) Percent area of ICAM-1 or VCAM-1 in GLUT1^+^ vessels. Each circle represents one mouse. *n =* 5–6 mice per group. **H-J**) Intravital 2-photon imaging was performed through a cranial window on eGFP-Claudin-5 mice injected with PBS or *T. gondii*. Longitudinal imaging was performed at baseline (0 dpi) and at 6 dpi in both control and infected mice. **H**) Representative field of view showing blood vessels in the cortex at both timepoints. Numbers indicate the same endothelial cells across days. Open arrowheads represent protrusions that were detected at 6 dpi. Scale bars, 20 μm. **I**) Endothelial cell protrusions at day 0 and day 6. A minimum of 3 vessel sections were quantified for each mouse across at least 3 fields of view (*n* = 32–52 cells total per mouse). **J**) Representation of shape index: circle has the highest shape index, 1, and a more tortuous shape has lower shape index. **K**) Shape index of brain endothelial cells at day 0 and day 6. Arrowheads point out protrusions. Each dot represents a single endothelial cell. *n =* 150–255 cells per group from 3–4 independent mice per group. **P < 0.005, *** *P* < 0.001, ****P < 0.0001; Student’s t test (**C**, **F**-**G**), one-way ANOVA (**I**-**J**). All error bars represent SD

To track *T. gondii* infection at the blood-brain barrier (BBB) and in the brain during acute infection, we utilized transgenic mice that express a fusion of eGFP to the tight junction protein Claudin-5 under the control of the Tie2 promoter (Tie2::eGFP-Claudin-5) to visualize the cerebral blood vessels [28, 36]. The mice were infected intraperitoneally with tdTomato-expressing type II *T. gondii* (*Prugniaud* strain) or mock-treated with PBS. Brain sections from mock and infected mice were imaged by confocal fluorescence microscopy during acute infection. *T. gondii* was detectable within the CNS blood vessels (Fig. 1A) and in the brain (Fig. 1B) during acute timepoints (5–9 days post-infection, dpi), demonstrating early dissemination of the parasite into the brain during infection. Detection of the *T. gondii*-specific gene B1 by qPCR revealed significantly more parasite DNA in the brains of infected mice than mock-treated mice at 9 dpi (Fig. 1C).

*T. gondii* infection induces a systemic inflammatory response [41–44] that influences the activation state of the vascular endothelium [45, 46]. Indeed, the levels of IFN-γ were significantly elevated by 2 dpi in *T. gondii-*infected mice compared to mock-treated mice and continued to be detected at high levels throughout the acute infection (Supplemental Fig. 1A). IL-12 was significantly upregulated at 4 dpi and then returned to baseline levels (Supplemental Fig. 1B). TNF-α increased throughout acute infection and was significantly elevated in infected mice by 9 dpi (Supplemental Fig. 1C). Consistent with the increased proinflammatory cytokines detected in the serum of infected mice, activation markers were upregulated in the brain endothelium: by staining brain sections from mock-treated or infected mice with anti-GLUT1 to delineate blood vessels (Fig. 1D-E, Supplemental Fig. 2A-B), we confirmed increased ICAM-1 and VCAM-1 vessel coverage in the brains of infected compared to mock-treated mice (Fig. 1F-G). These findings are consistent with prior studies demonstrating cell adhesion molecule upregulation in brain vascular endothelium during *T. gondii* infection [47–49] and indicate that our model aligns with previous models of *T. gondii* CNS infection. Notably, ICAM-1 and VCAM-1 signal were detected in infected mice in regions both with and without *T. gondii* parasites, indicating a global effect of infection on endothelial cell activation, likely due to systemic inflammation-induced upregulation of these adhesion molecules (Supplemental Fig. 2).

To investigate dynamic changes in the vascular endothelium during *T. gondii* infection, we conducted 2-photon imaging of the brain in the eGFP-Claudin-5 mice during acute infection. These mice enable the detection of changes in vascular organization both at the level of the vessel and at the level of individual endothelial cells, since each endothelial cell border is delineated with eGFP-Claudin-5 signal. With this imaging approach, we can image to 400 μm depth in the brain and return to the same imaging fields of view (FOVs) over time during infection. In conducting this longitudinal imaging, we found that the endothelial cell borders in the infected mice became profoundly distorted compared to the control PBS-injected mice, which underwent the same surgery and imaging procedures (Fig. 1H). We also noted increased numbers of protrusion structures in the Claudin-5 signal, specifically in *T. gondii*-infected mice (Fig. 1H-I, orange arrowheads). These protrusions have been previously reported and indicate active tight junction remodeling [28].

To quantify the changes in the endothelial cell morphology, we examined the shape index of the cells, which is used to monitor the roundness of endothelial cells (Fig. 1J): a circle has the highest shape index (of 1), and the more tortuous the cell, the lower the shape index [37]. At baseline, both groups of mice had similar shape index values (Fig. 1K). However, by 6 dpi, the shape index of cells decreased in the *T. gondii*-infected mice, reflecting the distorted shape of the endothelial cells over time. This change was not observed in the PBS-injected mice (Fig. 1K), indicating that this effect resulted from infection and not due to the surgery or imaging procedure. Similar to the endothelial cell activation detected by elevated adhesion molecule expression, the endothelial shape changes were observed in regions with and without *T. gondii*, further indicating a global effect of infection on the cerebral vasculature at the cellular level.

**Fig. 2:**
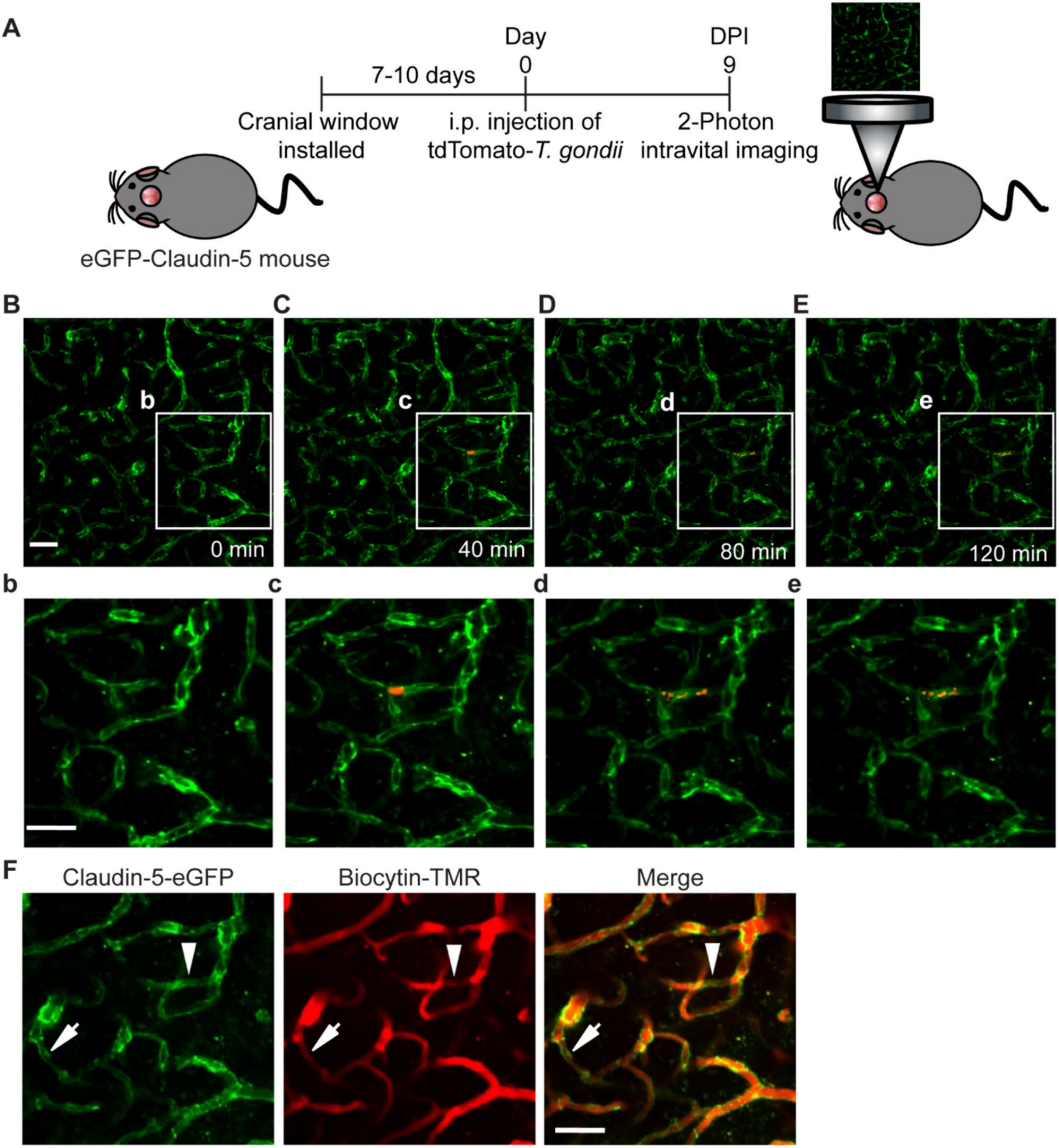
Reduced blood vessel perfusion during acute *T. gondii* infection. **A**) Schematic of experimental workflow for intravital 2-photon imaging in the cortex of an eGFP-Claudin-5 mouse infected with tdTomato-expressing *T. gondii* at 9 dpi. **B**-**E**) Imaging in the same field of view (FOV) at 0, 40, 80, and 120 min. **b-e**) Insets show a magnified FOV around the *T. gondii* vacuole. **F**) Biocytin-TMR (860 D) was injected i.v. during imaging and dye perfusion (red) was imaged in the same FOV as in **B-E**. Arrowhead shows the reduced dye perfusion adjacent to the *T. gondii *parasites. Arrow shows a location distal to the parasites with reduced perfusion. Scale bars, 50 μm

To examine more directly the effect of *T. gondii* infection on the BBB, we imaged infected eGFP-Claudin-5 mice for regions with detectable parasites by 2-photon microscopy (Fig. 2A, Supplementary Movie 1). Longitudinal imaging of mice infected with tdTomato-expressing *T. gondii* revealed a large vacuole of parasites in a cortical capillary at 9 dpi (Fig. 2C). Notably, this vacuole was not observed 40 min earlier (Fig. 2B), suggesting that it arrived at the capillary level within an infected cell circulating in the bloodstream. At 80 min after the start of imaging, the vacuole underwent spontaneous lysis, and individual tachyzoites could be detected (Fig. 2D), remaining in the same location 40 min later (Fig. 2E). After 120 min of imaging (Fig. 2F), biocytin-TMR, an 860 Da fluorescent dye, was injected intravenously to determine the effect of this vacuole lysis event on vessel injury or perfusion. Interestingly, we did not detect leakage of this very low-molecular-weight dye near the region of parasites or elsewhere in the FOV, suggesting an intact BBB. However, we did detect apparent vessel occlusion near the parasites. Scanning through the z-stacks revealed that the region of the blood vessel immediately adjacent to the site of parasite lysis was not perfused with dye (Fig. 2F, white arrowhead; Supplementary Video 1). We also detected regions not immediately adjacent to parasites, which also appeared to have reduced dye perfusion (Fig. 2F, white arrow; Supplementary Video 1). These data suggested that *T. gondii* infection leads to changes in the morphology of endothelial cells. Moreover the reduced perfusion of vascular dyes in cerebral blood vessels, both near parasites and at more distant sites, indicated that acute *T. gondii* infection resulted in an obstruction of blood flow.

### *T. gondii* infection induces clotting in the brain microvasculature

**Fig. 3:**
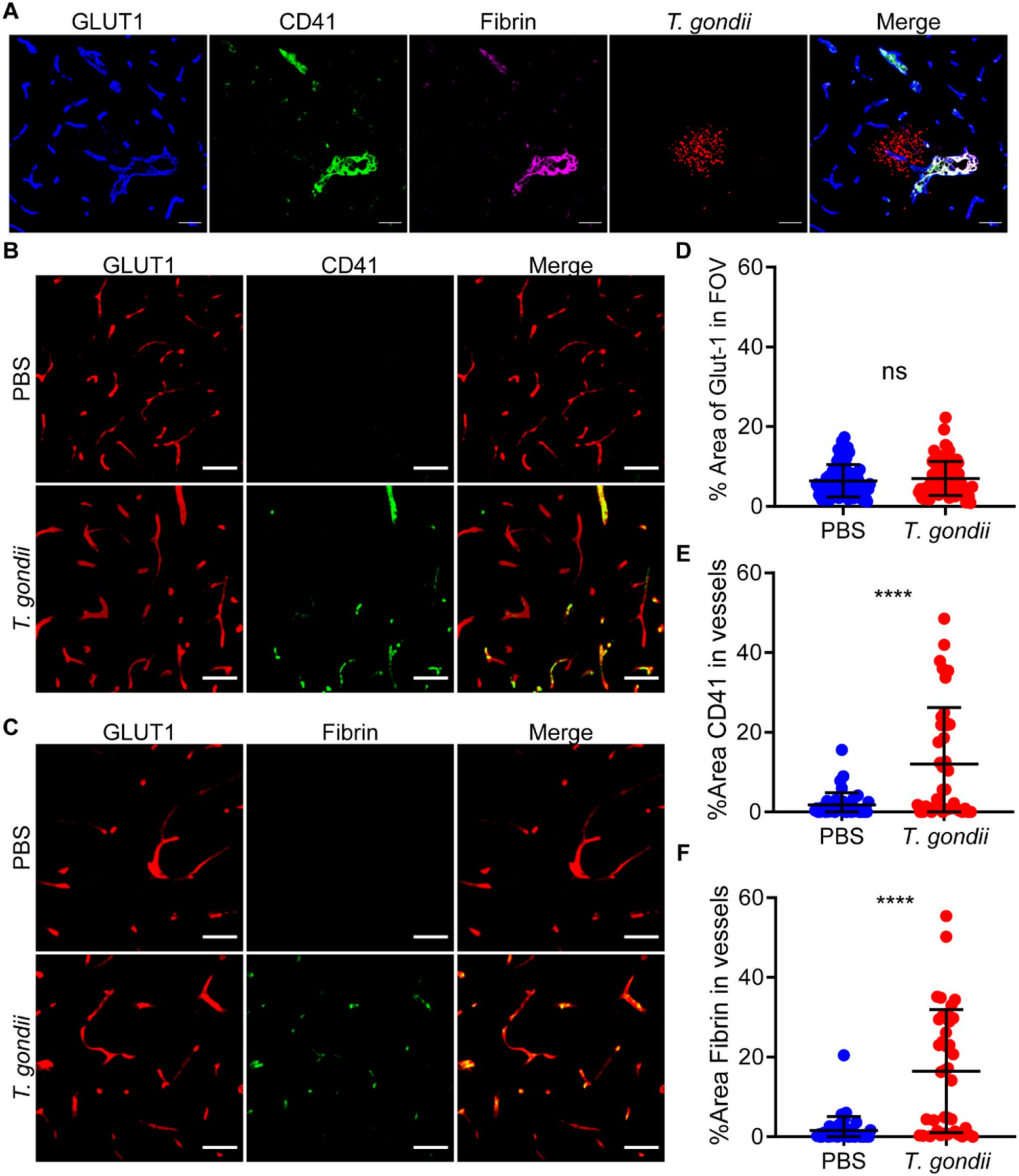
Evidence of platelet-fibrin clot formation in cerebral vessels of *T. gondii*-infected mice. C57BL/6J mice were infected with *T. gondii* or injected with PBS, and brains were harvested at 9 dpi. **A**) Confocal image of brain section stained with antibodies against GLUT1, CD41, and fibrin. Scale bars, 50 μm. **B**-**C**) Widefield images of brain sections stained with anti-GLUT1 and either anti-CD41 (**B**) or anti-fibrin (**C**). Scale bars, 50 μm. **D**) Percent area of GLUT1 within each FOV. **E**-**F**) Percent area of CD41 or fibrin in GLUT1^+^ vessels, respectively. Each circle represents one FOV. *n* = 40–80 FOVs from 4 independent mice per group. **** *P* < 0.0001; Student’s t test. Error bars represent SD

One possible explanation for the reduced dye perfusion in the brains of *T. gondii-*infected mice is the formation of blood clots in the cerebral vessels. To investigate this possibility, we examined the end products of the clotting cascade: platelets and fibrin. Indeed, the deposition of CD41^+^ platelets and fibrin within GLUT1^+^ blood vessels was detectable near large sites of parasite infection (Fig. 3A). Optical clearing [34] enabled us to image GLUT1, fibrin, and CD41 throughout the cortex of mock and *T. gondii*-infected mice (Fig. 3B-C). GLUT1 signal was not significantly different between the mock and infected mice over several FOV per infected animal (Fig. 3D). However, the percent area of CD41 and fibrin in GLUT1^+^ vessels was significantly increased in the brains of infected compared to mock-treated mice (Fig. 3E-F). Interestingly, the CD41 and fibrin deposition was frequently detectable in infected brains in FOVs without parasitic foci. These data indicate that acute *T. gondii* infection leads to the formation of platelet-fibrin clots in the cerebral vasculature, both near *T. gondii* and in regions without detectable parasites.

### Hemodynamic changes during *T. gondii* infection

**Fig. 4:**
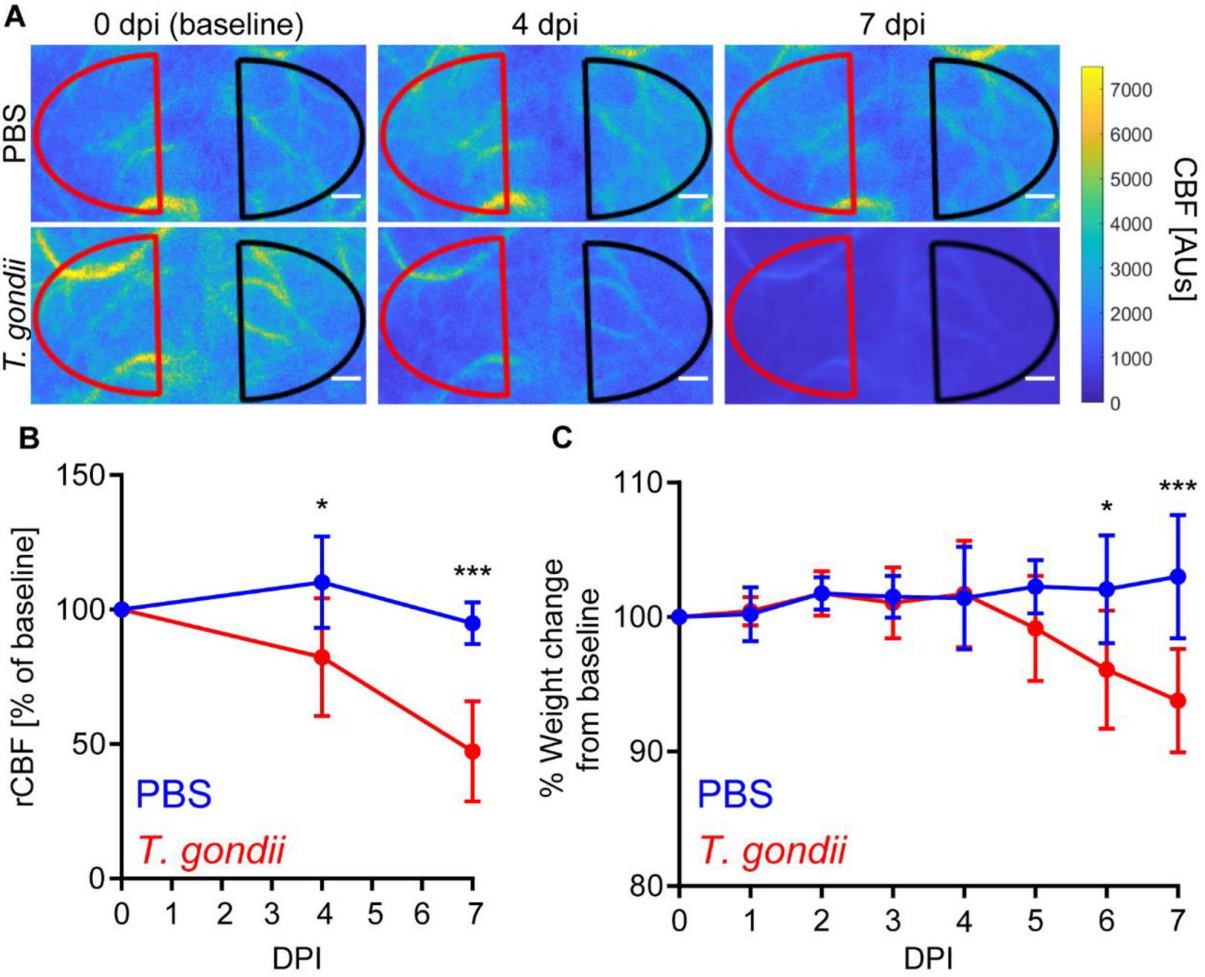
CBF changes during acute *T. gondii* infection. C57BL/6 mice were injected with PBS or infected with *T. gondii* and longitudinal laser speckle imaging was performed at 0, 4, and 7 dpi through the intact skull to measure CBF. **A**) Representative laser speckle images of control and *T. gondii*-infected mice. Scale bars, 400 μm. **B**) Percent change of rCBF to baseline in control and *T. gondii*-infected mice. **C**) Percent weight change from baseline in control and *T. gondii*-infected mice. *n* = 4–5 mice per group. *P < 0.05, ***P < 0.001; significance between mock and infected mice at each timepoint was calculated by a repeated measures two-way ANOVA, with a post hoc Sidak’s multiple comparisons test. Error bars represent SD

Due to the increased tortuosity of cerebral endothelial cells and the detection of coagulation at the BBB, we next investigated the degree to which acute *T. gondii* infection resulted in cerebral hemodynamic changes. We recently developed a cyanoacrylate skull surgery in mice [38], and we used this technique coupled with laser speckle imaging (LSI) to measure CBF through the intact skulls of C57BL/6J mice. This approach allowed us to image the same mice longitudinally from acute to chronic infection, starting with baseline measurements at 0 dpi, prior to injection with either PBS or *T. gondii* (Fig. 4A). We found that relative CBF (rCBF) in PBS-treated mice was stable. However, in *T. gondii-*infected mice, rCBF significantly decreased by 4 dpi, and the decline became more profound by 7 dpi (Fig. 4B). Interestingly, the reduction in rCBF preceded weight loss induced by *T. gondii*-infection, as the weight changes of *T. gondii*-infected mice were only significantly different than the mock-treated mice at 6 dpi (Fig. 4C).

**Fig. 5:**
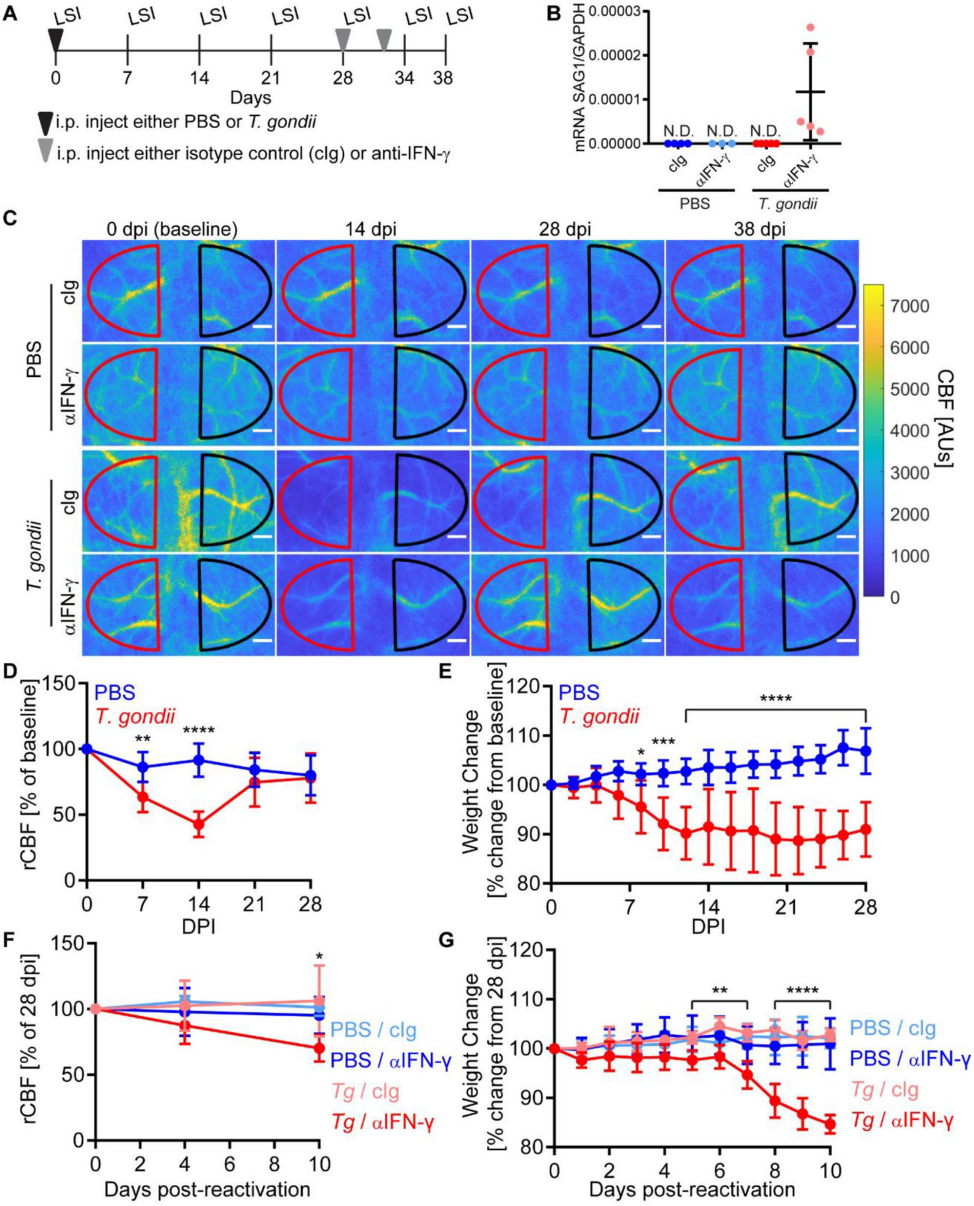
CBF changes during chronic *T. gondii* infection. **A**) Experimental set-up for chronic infection and reactivation laser speckle imaging (LSI) experiments. C57BL/6 mice were injected with PBS or infected with 200 type II *T. gondii* tachyzoites at 0 dpi after the first imaging session. Antibodies were injected at 28 and 32 dpi. **B**) Relative expression of SAG1 to GAPDH transcripts in brains of control (PBS) and *T. gondii*-infected mice at 38 dpi. Mice were administered control IgG (cIg) or anti-IFN-γ (αIFN-γ) starting at 28 dpi. SAG1 was not detected (ND) in any group except the *T. gondii*-infected mice given anti-IFN-γ. **C**) Representative laser speckle imaging showing CBF in control (PBS) or *T. gondii*-infected mice at 0, 14, 28, and 38 dpi. The red and black hemispheres show the ROIs for the right and left hemispheres. Scale bars, 400 μm. **D**) Percent change of rCBF to baseline in control and *T. gondii*-infected mice over time. **E**) Percent weight change from baseline in control and *T. gondii*-infected mice. (**F**) Percent change of rCBF in control and *T. gondii*-infected mice (*Tg*) administered cIg or anti-IFN-γ. (**G**) Percent weight change in control and *T. gondii*-infected mice administered cIg or anti-IFN-γ during reactivation. *n* = 8–11 mice (**D**-**E**) and 4–7 mice per group (**F**-**G**). *P < 0.05, **P < 0.005, ***P < 0.001, ****P < 0.0001; differences between groups at each timepoint were determined by a two-way ANOVA (**D**-**E**) or a mixed-effects analysis (**F**-**G**) with a post-hoc Sidak’s multiple comparisons test. In **F-G**, significance is shown between *T. gondii*-infected mice treated with IgG or anti-IFN-γ. Error bars represent SD

We next investigated hemodynamic changes over the entire course of infection (acute, chronic, and reactivation stages) by infecting C57BL/6J mice with a lower dose of *T. gondii* and tracking the mice to 38 dpi (Fig. 5A). Since IFN-γ is an essential mediator of host defense against *T. gondii* [50], the administration of anti-IFN-γ mAb to the mice during the chronic stage of infection neutralizes the cytokine and induces reactivation of chronic infection both in the brain and the periphery [51]. Anti-IFN-γ was administered at 28 and 32 dpi, and reactivation was confirmed by the elevated levels of SAG1 transcripts detected at 38 dpi in the brains of infected mice injected with anti-IFN-γ, but not in infected mice injected with the control Ig (cIg) (Fig. 5B). Longitudinal LSI was conducted on these mice, and representative images from the 0, 14, 28, and 38 dpi timepoints are shown (Fig. 5C). Consistent with the acute infection data (Fig. 4A and B), infected mice had a significant decrease in CBF by 14 dpi compared to PBS-treated mice, but rCBF recovered to the level of the PBS-treated mice by 21 dpi (Fig. 5C and D). Notably, the changes in rCBF were distinct from the weight changes in the *T. gondii*-infected mice, which did not regain their pre-infection weight even in chronic infection (Fig. 5E). During the reactivation of infection induced by anti-IFN-γ administration, there was again a significant decrease in rCBF, which was not detected in chronically-infected mice injected with the isotype control antibody (Fig. 5F). Consistent with the reactivation of infection, this group also experienced significant weight loss (Fig. 5G). Collectively, these data demonstrate a reduction in CBF during acute and reactivated *T. gondii* infection, and that the mice recover normal levels of CBF during stable chronic infection.

**Fig. 6:**
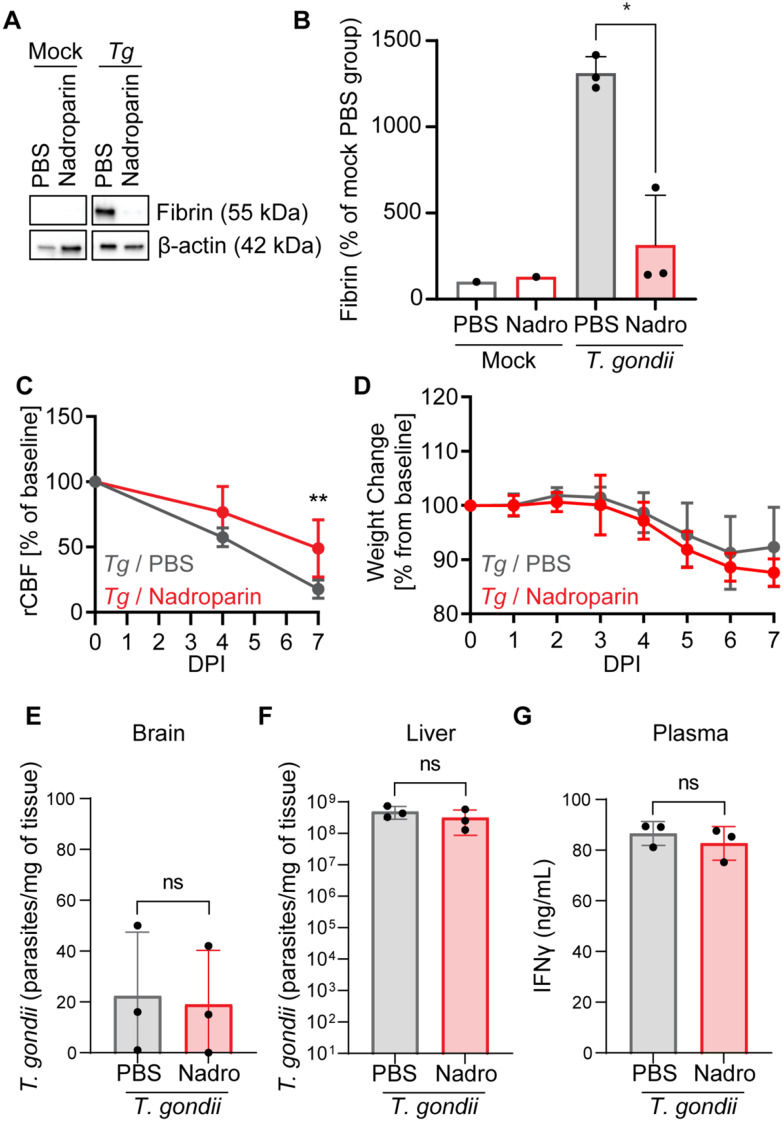
Nadroparin calcium treatment of *T. gondii*-infected mice. **A**) Representative Western blot of fibrin and β-actin from livers of mock- and *T. gondii-*infected mice treated with PBS or Nadroparin at 7 dpi. **B**) Quantification of fibrin in mock-treated mice given PBS or nadroparin and *T. gondii*-infected mice given PBS or nadroparin. *n* = 1–3 mice per group. * *P* < 0.05; significance was calculated by one-way ANOVA followed by a Tukey post-test. **C**) Percent change of rCBF to baseline in *T. gondii*-infected mice treated with PBS or nadroparin calcium at 0, 4, and 7 dpi. **D**) Percent weight change from baseline in *T. gondii*-infected mice treated with PBS or nadroparin calcium. *n* = 3–6 mice per group. *P < 0.05, **P < 0.005, ns = not significant; significance was calculated by a mixed-effects analysis with a post hoc Sidak’s multiple comparisons test. Brain (**E**) and liver (**F**) homogenates were examined for the *T. gondii* B1 gene by qPCR to determine *T. gondii* per mg of tissue in *T. gondii*-infected mice treated with PBS or nadroparin (7 dpi). **G**) IFN-γ levels were quantified with an ELISA in *T. gondii*-infected mice treated with PBS or nadroparin (7dpi). *n* = 3 mice per group. Significance was calculated with a Student’s *t* test. Error bars represent SD

Finally, we assessed the contribution of clotting to CBF changes by treating *T. gondii*-infected mice with a low-molecular-weight heparin, nadroparin calcium, and measuring CBF longitudinally. Both the intrinsic and extrinsic clotting pathways converge on factor X activation (factor Xa), which cleaves prothrombin to thrombin. Low-molecular-weight heparins inhibit clotting by binding to antithrombin and enhancing its enzymatic activity and inhibition of factor Xa [52]. We first verified that nadroparin treatment reduced fibrin deposition in livers of infected animals (Fig. 6A-B). Longitudinal laser speckle imaging was performed at 0, 4, and 7 dpi on infected mice treated with nadroparin or vehicle control. We observed a partial rescue of rCBF in *T. gondii*-infected mice treated with nadroparin calcium compared to those treated with PBS at 7 dpi (Fig. 6C). Interestingly, there was not a significant difference in weight loss between these groups (Fig. 6D), nor was there a significant difference in parasite dissemination in either the brain or the liver (Fig. 6E-F), or in systemic IFN-γ production (Fig. 6G). Finally, we analyzed whether the composition of immune cells was different in the blood, brains and livers of infected mice treated with nadroparin (Supplemental Figs. 3–5) using flow cytometry. We found significantly reduced CD45^+^ cells in the blood (Supplemental Fig. 3B-C), no significant changes in the immune cells in the brain (Supplemental Fig. 4B-C) and increased B cells in the livers of nadroparin-treated infected mice (Supplemental Fig. 5B-C). Collectively, these data suggest that clotting contributes to decreased CBF during acute *T. gondii* infection without affecting parasite burden in the liver or brain.

## Discussion

The term ‘immunothrombosis’ was recently coined by Engelmann and Massberg [12] and refers to an innate immune response induced by the coagulation cascade in blood vessels. In the context of immunothrombosis, coagulation supports the accumulation of myeloid cells, such as neutrophils and monocytes, at sites of infection, as well as the activation of the complement cascade [12]. These processes enhance the ability of the host to limit pathogen dissemination and control the infection (i.e., *E. coli* and *Yersinia pestis*) [4, 9].

**Fig. 7:**
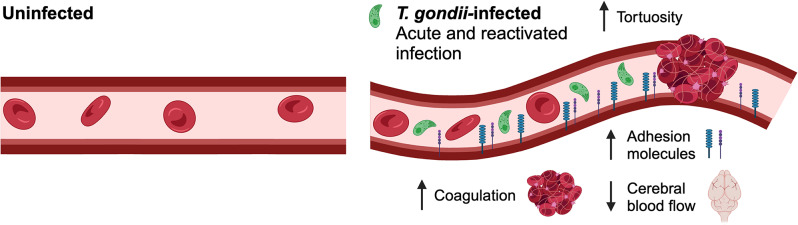
Model of changes to the brain microvasculature during *T. gondii* infection. On the left is the brain microvasculature of an uninfected mouse, with no upregulation of adhesion molecules, and no changes to tortuosity. On the right, we demonstrate that in the brain microvasculature of *T. gondii*-infected mice there is increased tortuosity and adhesion molecules (VCAM-1 and ICAM-1), as well as increased coagulation, resulting in decreased cerebral blood flow (created using Biorender)

Prior research by Luo et al. and Mullarky et al. demonstrated evidence for activation of the clotting cascade in the livers of mice during *T. gondii* infection [53, 54]. These studies showed an increase in insoluble fibrin in the livers of *T. gondii*-infected mice, which is highly suggestive that thrombosis has occurred. Notably, coagulation in the brain vasculature has not previously been examined in *T. gondii*-infected mice. Moreover, the vascular beds in the liver and the brain are highly different: the liver has fenestrated endothelium, which is relatively “leaky,” whereas the tight junctions that comprise the neurovascular unit result in the formidable blood-brain barrier [55–58]. Our current findings build on the research of Smiley and colleagues to show direct evidence of platelet-fibrin clots in the cerebral vasculature during acute infection. The observation that the anticoagulant nadroparin partially rescued cerebral blood flow during infection suggests that clot formation in the brain vasculature functionally reduced cerebral hemodynamics. Consistent with prior studies, we also observed increased endothelial cell activation and increased blood vessel tortuosity. These effects on the endothelium correlated with high levels of proinflammatory cytokines during the acute phase of infection. We propose a model in which systemic *T. gondii* infection increases circulating proinflammatory cytokines, which upregulate ICAM-1 and VCAM-1 expression and vessel tortuosity. These features can prime the endothelium, such that *T. gondii* infection and lysis of endothelial cells in the cerebral vasculature may then trigger clot formation and subsequent reduced cerebral blood flow (Fig. 7).

Although the formation of blood clots may prove beneficial for ensnaring some pathogens in the bloodstream, clotting in the cerebral vasculature has the potential to be detrimental. This may be the case in COVID-19 patients, some of whom have evidence of both cerebral ischemic stroke and reduced CBF [11, 17]. Recent research has linked the accumulation of neutrophil extracellular traps (NETs) in the brain microvasculature to this pathology [59]. Furthermore, evidence of clotting is associated with negative survival outcomes in pediatric cerebral malaria cases [13–16]. The current study demonstrates evidence of coagulation in cerebral vessels during infection with *T. gondii*. Although *Toxoplasma* and *Plasmodium* are both apicomplexan parasites that share many structural and biochemical features, the mechanisms of pathogenesis caused by these two pathogens are distinct. Indeed, in cerebral malaria, thrombosis is associated with regions of infected erythrocytes sequestered in small vessels in the brain due to cytoadhesion [13]. In particular, the virulence of *P. falciparum* infection is linked to the ability of the parasite-infected RBCs to form “knobs,” which are nanoscale-sized protrusions on the surface of infected erythrocytes. These knobs are comprised of knob-associated histidine-rich protein (KAHRP) and *P. falciparum* erythrocyte membrane protein 1 (PfEMP1), and they facilitate the binding of infected erythrocytes to the vascular endothelium [10, 60, 61].

Unlike in cerebral malaria, clotting during *T. gondii* infection was detected at sites in the vasculature both near and far from clusters of *T. gondii* parasites. Previous research has found an effect of *T. gondii* infection on cerebral blood flow, which was attributed to the pruning of the cerebral microvessels as a consequence of infection [23]. Interestingly, in this model of infection reported by Estato et al., the reduced CBF was detected during acute infection (10 dpi) and at 40 dpi, only recovering by 180 dpi [38]. Consistent with this work, we also observed decreased CBF during acute infection. Our study extends the prior findings of Adesse and colleagues by using the surgical technique we developed to longitudinally monitor CBF in the same mice from acute to chronic infection. We also made the new observation that parasite reactivation due to depletion of protective IFN-γ resulted in a similar reduction in CBF as observed during acute infection. Collectively, these data suggest that the host response to tachyzoite replication during acute infection and reactivated infection can influence cerebral hemodynamics. Given that the inflammatory environment is markedly different during these lytic infection stages, as the immune system responds to the rapidly dividing parasites, it is likely that the reduced CBF is due to a complex series of events that accompany host defense against the parasite.

Notably, we observed a partial rescue of CBF with anticoagulation therapy. These findings indicate that other mechanisms also contribute to the reduced CBF during *T. gondii* infection. One possibility is that the reduced CBF may relate to the architecture of the cerebral vasculature. Indeed, Estato et al. reported evidence of microvessel pruning, and we detected increased vessel and endothelial cell tortuosity during acute infection. Increased endothelial cell tortuosity has been associated with changes in blood pressure, which cause re-arrangement of the cytoskeleton of endothelial cells [37, 62, 63]. Vessel tortuosity has been linked to decreased CBF during aging, potentially explaining the decrease in CBF observed during *T. gondii* infection [64]. Future studies will be needed to determine the other factors contributing to decreased cerebral blood flow in *T. gondii* infection.

One outstanding question is the cause of clotting during *T. gondii* infection. In cases of clotting near foci of infection, it is possible that an infected endothelial cell has recently lysed, exposing subendothelial tissue factor (TF) and initiating an intravascular clotting event. Another possibility is that myeloid cells upregulate TF during *T. gondii* infection in vivo and trigger coagulation in the sites where they accumulate. Past work has shown that monocytes and neutrophils exposed to LPS can upregulate TF [3, 5, 6, 65, 66]. We and others have previously found that monocytes accumulate in the cerebral vessels of *T. gondii*-infected mice [30, 38], which may explain the coagulation at sites distant from foci of infection. These large numbers of adherent monocytes in the blood vessels could also contribute to the formation of clots by causing more turbulent blood flow, which creates an environment more favorable to clot formation. Coagulation could also be initiated through the intrinsic pathway of coagulation if activated platelets or other negatively charged surfaces are exposed to Factor XII within plasma.

It remains to be determined whether thrombosis during *T. gondii* infection is beneficial to the host or detrimental. As previously mentioned, thrombosis can be an important facet of host defense against pathogens (as has been shown with *E. coli* and *Y. pestis*) as a mechanism of ensnaring pathogens and physically preventing them from disseminating. In addition, thrombosis creates a compartment in which antimicrobial peptides accumulate and also generates an environment in which additional immune cells can rapidly aggregate [4, 9]. However, our work presented here found no difference in parasite burden in the liver or brains of anti-coagulated *T. gondii*-infected mice within the timeframe of our analysis (7 dpi). We did detect a decrease in CD45^+^ cells in the blood of nadroparin-treated *T. gondii*-infected mice, though further studies are needed to investigate whether this is due to a change in the production of CD45^+^ cells or whether they are recruited to a site we did not probe during infection. Interestingly, we also found that B cells were increased in the livers of anti-coagulated *T. gondii*-infected mice, though implications of this finding require further investigation, as there is no clear link between thrombosis and B cell localization. Previous research has demonstrated that mice treated with warfarin and fibrinogen-deficient mice die during acute *T. gondii* infection compared to mock-treated or fibrinogen-sufficient control mice [67]. Future studies will be needed to determine whether clotting helps to protect against hemorrhage during *T. gondii* infection.

## Conclusions

We examined the effect of *Toxoplasma gondii*, a global foodborne parasite that establishes chronic infection in the CNS, on the blood-brain barrier and on cerebral blood flow during infection. Using a combination of fixed tissue and in vivo imaging of mice, we found that CNS endothelial cells upregulate adhesion molecules and become more distorted during infection. In addition, we detected evidence of coagulation (platelets and fibrin) during infection that reduced cerebral blood flow. These data suggest that changes in cerebral hemodynamics may be a common feature of inflammation and infection, particularly with CNS pathogens.

## Electronic supplementary material

Below is the link to the electronic supplementary material.

Supplementary Material 1

Supplementary Material 2

## Data Availability

No datasets were generated or analysed during the current study.

## Abbreviations

BBB: Blood-Brain Barrier
CBF: Cerebral Blood Flow
cIg: Control Ig
CNS: Central Nervous System
DPI: Days Post-Infection
FOV: Field Of View
i.p.: intraperitoneally
LSI: Laser Speckle Imaging
mAb: Monoclonal Antibody
rCBF: Relative Cerebral Blood Flow
ROI: Region Of Interest
TF: Tissue Factor
WT: Wild-Type
