# Supplementary material for: Infection with Toxoplasma gondii triggers coagulation at the blood-brain barrier and a reduction in cerebral blood flow
Source: J Neuroinflammation. 2025 Jan 8;22:3. doi: 10.1186/s12974-024-03330-1 (PMC11708167; doi:10.1186/s12974-024-03330-1)
Supplement: Supplementary file 2 — Supplementary Material 2 [file 12974_2024_3330_MOESM2_ESM.pdf]

Infection with *Toxoplasma gondii* triggers coagulation at the blood-brain barrier and a reduction in cerebral blood flow

Supplementary Information

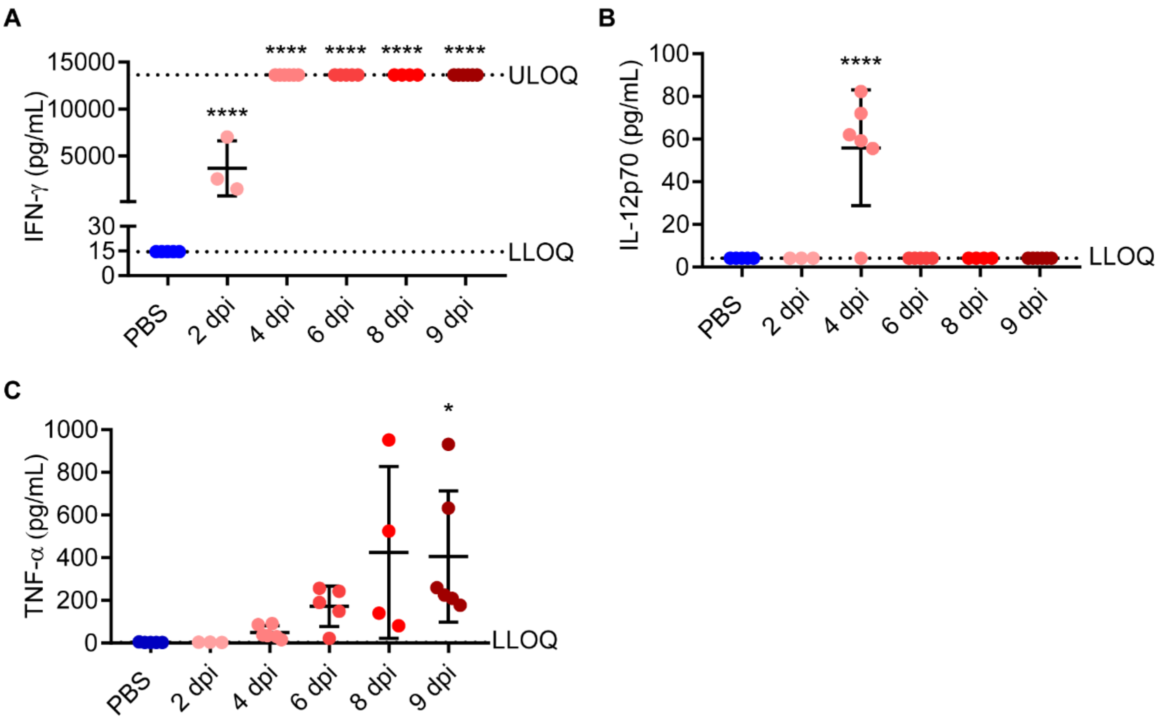

Supplemental Fig. 1. Cytokine Response During Acute *T. gondii* infection. Levels of IFN-γ (A), IL-12p70 (B), and TNF-α (C) in the serum of PBS-injected at 9 days post-injection or *T. gondii*-infected mice at the indicated time points, as measured by multiplex analysis. Each circle represents one mouse. Dotted lines indicate the upper or lower limit of quantification (ULOQ or LLOQ, respectively). *n*= 3-10 mice per group. *P < 0.05, ****P < 0.0001. Significance was calculated by a one-way ANOVA with a post-hoc Tukey test. All error bars represent SD.

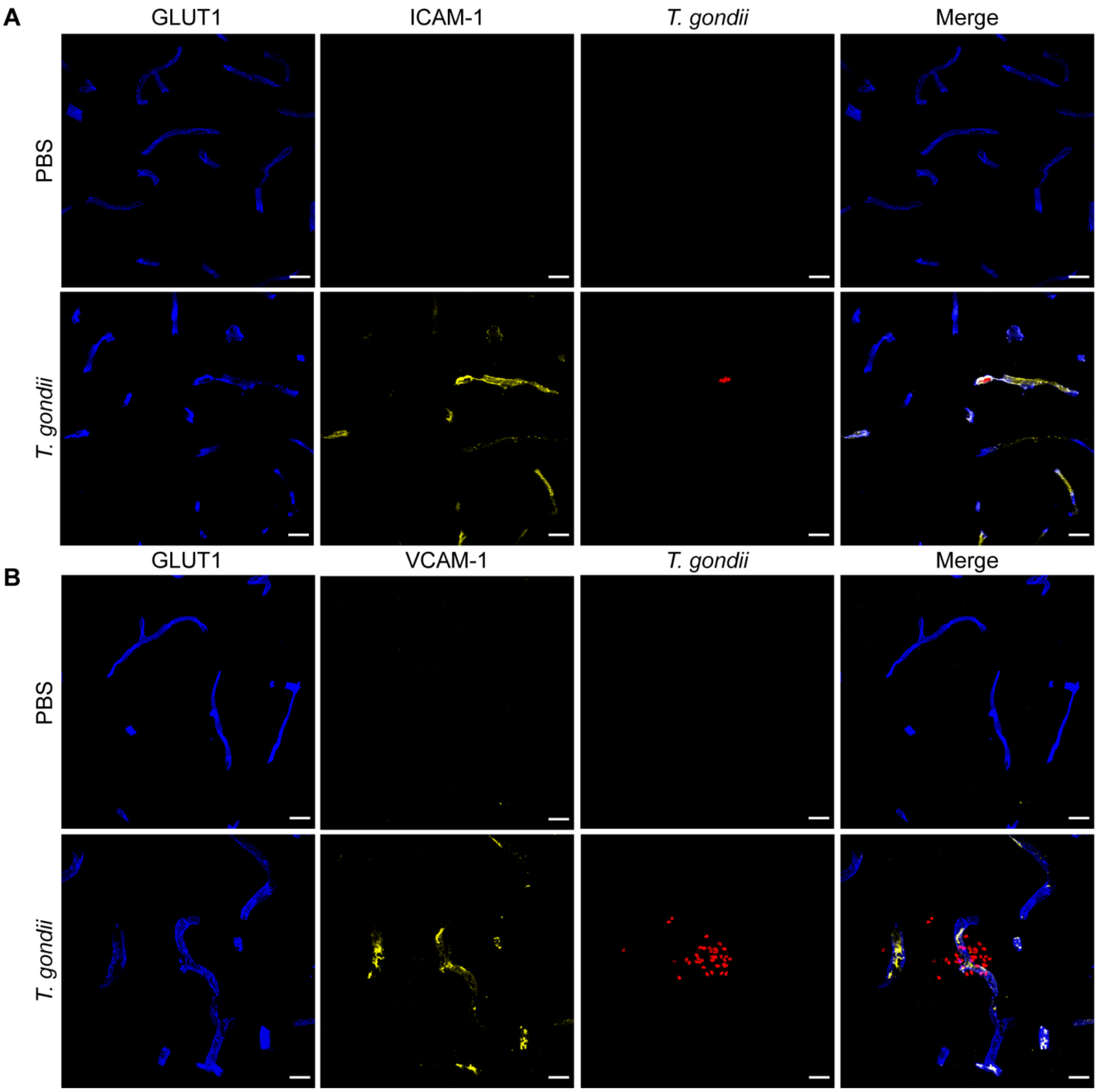

Supplemental Fig. 2. Individual GLUT1, ICAM-1, VCAM-1, and *T. gondii* staining showing increased endothelial adhesion molecule expression in *T. gondii*-infected mice. C57BL/6J mice were injected with PBS or infected with *T. gondii,* and brains were harvested at 7 dpi. A-B) Representative confocal images of brain sections stained with antibodies against GLUT1 and (A) ICAM-1 or (B) VCAM-1. Scale bars, 15 µm. These images show the unmerged FOVs represented in Figure 1D.

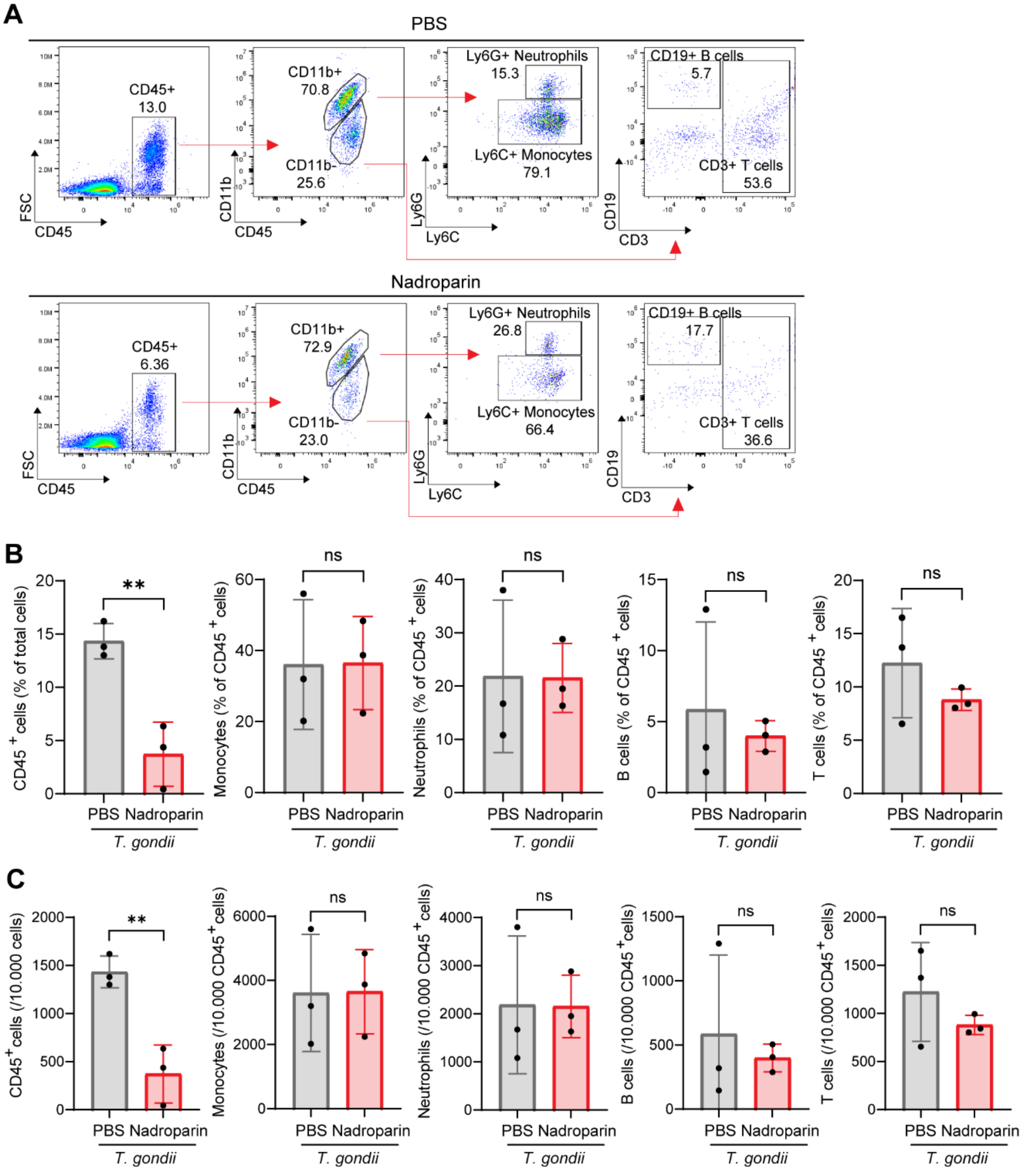

Supplemental Fig. 3. Immune cell phenotypes in the blood of *T. gondii*-infected mice after treatment with nadroparin. A) Representative gating of CD45+ cells from the blood of PBS-treated or nadroparin treated *T. gondii*-infected mice. B) Frequency of immune cells in the blood of PBS-treated or nadroparin treated *T. gondii*-infected mice at 7 dpi. C) Absolute cell numbers of immune cells per 10,000 cells in the blood of PBS-treated or nadroparin treated *T. gondii*-infected mice at 7 dpi. n = 3 mice per group (the same mice are shown in B and C). **P < 0.005, ns=not significant; significance was calculated with a Student's t test. Error bars represent SD.

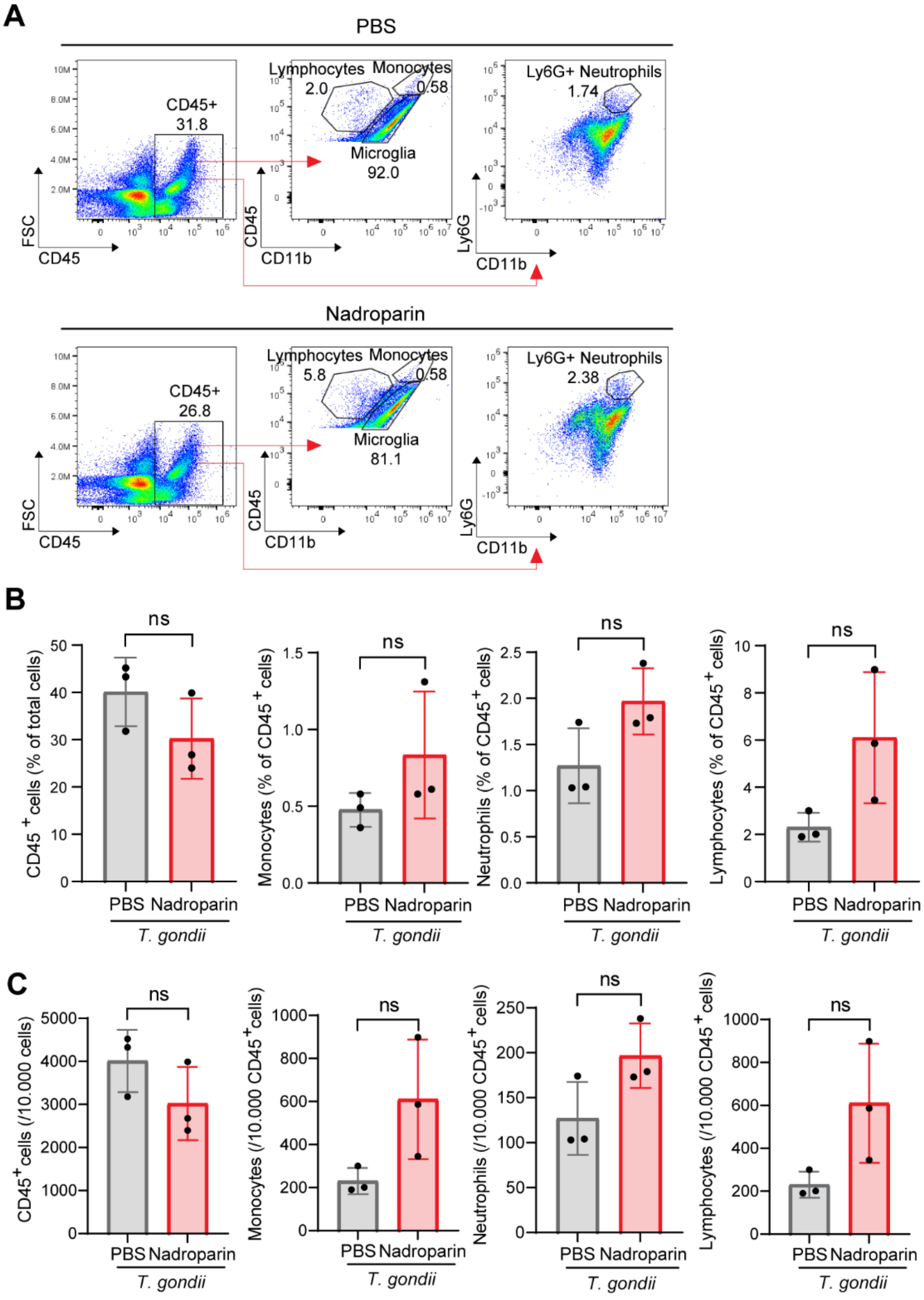

Supplemental Fig. 4. Immune cell phenotypes in the brains of *T. gondii*-infected mice after treatment with nadroparin. A) Representative gating of $CD45^+$ cells from the brains of PBS-treated or nadroparin treated *T. gondii*-infected mice. B) Frequency of immune cells in brains of PBS-treated or nadroparin treated *T. gondii*-infected mice at 7 dpi. C) Absolute cell numbers of immune cells per 10,000 cells in brains of PBS-treated or nadroparin treated *T. gondii*-infected mice at 7 dpi. n = 3 mice per group (the same mice are shown in B and C). ns=not significant; significance was calculated with a Student's t test. Error bars represent SD.

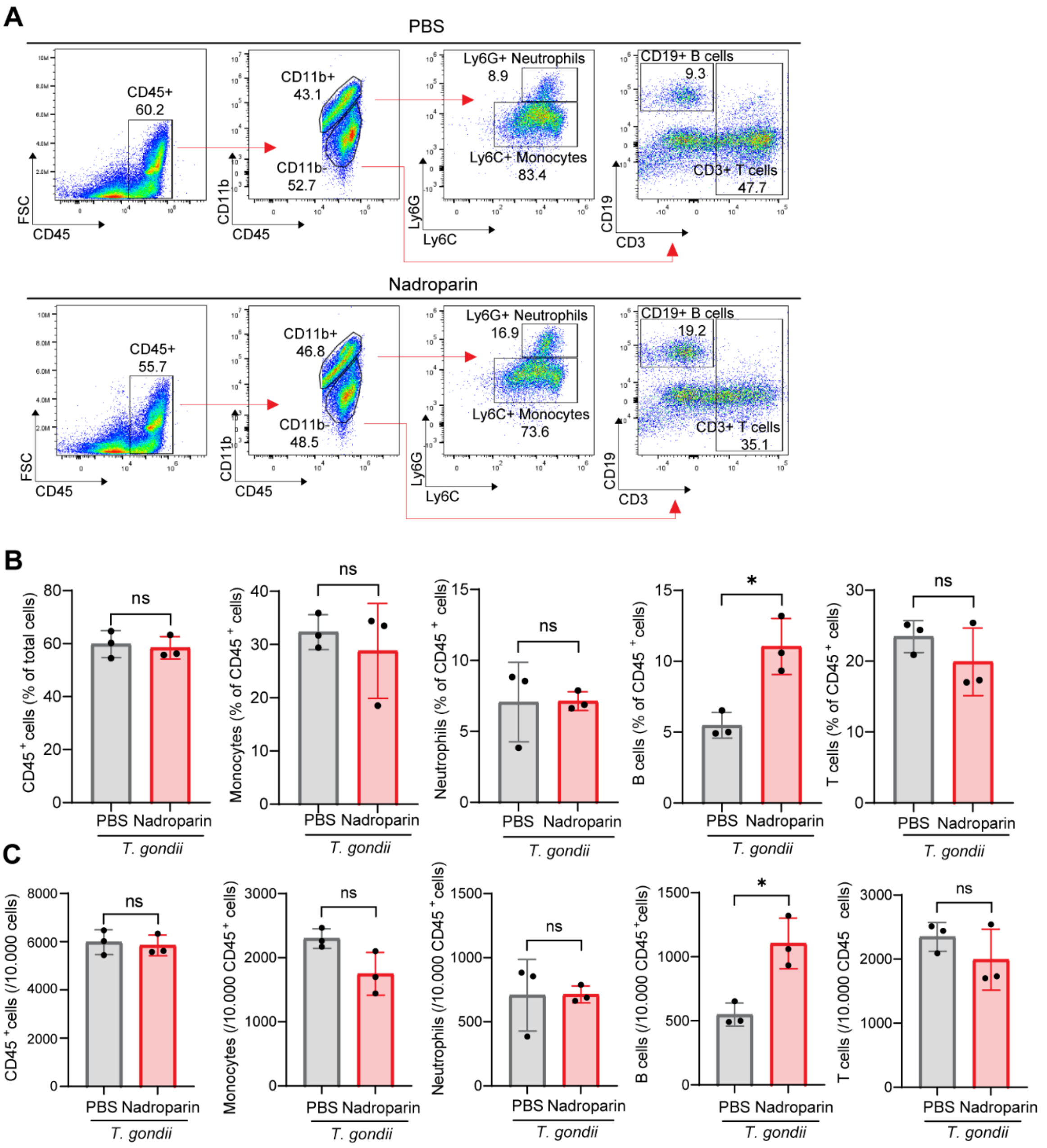

Supplemental Fig. 5. Immune cell phenotypes in the livers of *T. gondii*-infected mice after treatment with nadroparin. A) Representative gating of CD45$^+$ cells from the livers of PBS-treated or nadroparin treated *T. gondii*-infected mice. B) Frequency of immune cells in livers of PBS-treated or nadroparin treated *T. gondii*-infected mice at 7 dpi. C) Absolute cell numbers of immune cells per 10,000 cells in livers of PBS-treated or nadroparin treated *T. gondii*-infected mice at 7 dpi. n = 3 mice per group (the same mice are shown in B and C). *P < 0.05, ns=not significant; significance was calculated with a Student's t test. Error bars represent SD.

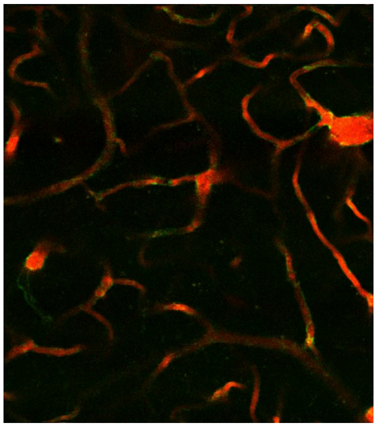

Supplemental Video 1. 2-Photon imaging showing reduced blood vessel perfusion during acute *T. gondii* infection. Intravital 2-photon imaging in the cortex of an eGFP-Claudin-5 mouse infected with tdTomato-expressing *T. gondii* at 9 dpi. Video shows the FOV, scanning through the z-planes, both before and after i.v. injection of Biocytin-TMR (red).
